# Correction: Gamification of Incentive Spirometry in Trauma Patients: Protocol for a Prospective, Observational Feasibility Study

**DOI:** 10.2196/83839

**Published:** 2025-09-26

**Authors:** Areen Al-Dhoon, Arthur Grimes, Carma Goldstein, Peter Spronk, R Shayn Martin, Aarti Sarwal

**Affiliations:** 1 Department of Neurology Atrium Health Wake Forest Baptist Medical Center Winston Salem, NC United States; 2 Department of Surgery, College of Medicine University of Oklahoma Oklahoma, OK United States; 3 Department of Surgery, Faculty of Medicine Atrium Health Wake Forest Baptist Medical Center Winston Salem, NC United States; 4 Department of Intensive Care, Expertise Center for Intensive Care Rehabilitation Apeldoorn Gelre Hospitals Apeldoorn The Netherlands; 5 Division of Neurocritical Care, Faculty of Medicine Virginia Commonwealth University Richmond, VA United States

In “Gamification of Incentive Spirometry in Trauma Patients: Protocol for a Prospective, Observational Feasibility Study” (JMIR Res Protoc 2025;14(1):e75871) the authors made two clarifications.

The Results section in the Abstract has been changed from:

The study was approved by the institutional review board of Atrium Health Wake Forest Baptist Medical Center and was registered at ClinicalTrials.gov. However, no participants were ever enrolled, as the OmniFlow partners did not supply the device for the study. Despite this, it remains a potentially beneficial tool, and future studies may confirm its benefits once the device becomes available.

to

The study was approved by the institutional review board and registered on ClinicalTrials.gov. Due to an unexpected delay in the initiation of the project, no patients were enrolled.

The Results section in the main body of the paper has been changed from:

The study was approved by the institutional review board and registered on ClinicalTrials.gov. However, no participants were enrolled, as the OmniFlow devices were never delivered. Despite initial agreements, the device supplier became unresponsive and failed to fulfill the shipment. Multiple attempts to reestablish contact were unsuccessful. This unexpected barrier highlights the importance of reliable vendor partnerships when implementing novel therapeutic technologies in clinical research.

to

The study was approved by the institutional review board and registered on ClinicalTrials.gov. Due to an unexpected delay in the initiation of the project, no patients were enrolled.

